# Antioxidant and Moisturizing Properties of Carboxymethyl Chitosan with Different Molecular Weights

**DOI:** 10.3390/polym12071445

**Published:** 2020-06-28

**Authors:** Nareekan Chaiwong, Pimporn Leelapornpisid, Kittisak Jantanasakulwong, Pornchai Rachtanapun, Phisit Seesuriyachan, Vinyoo Sakdatorn, Noppol Leksawasdi, Yuthana Phimolsiripol

**Affiliations:** 1 Faculty of Agro-Industry, Chiang Mai University, Chiang Mai 50100, Thailand; meen.nareekan@gmail.com (N.C.); kittisak.jan@cmu.ac.th (K.J.); pornchai.r@cmu.ac.th (P.R.); phisit.seesuriyachan@gmail.com (P.S.); design_by_yu@hotmail.com (V.S.); noppol@hotmail.com (N.L.); 2 Faculty of Pharmacy, Chiang Mai University, Chiang Mai 50200, Thailand; pimporn.lee@cmu.ac.th; 3 Cluster of Agro Bio-Circular-Green Industry, Chiang Mai University, Chiang Mai 50100, Thailand; 4 Center of Excellence in Materials Science and Technology, Faculty of Science, Chiang Mai University, Chiang Mai 50200, Thailand

**Keywords:** chitosan, carboxymethyl chitosan, molecular weight, antioxidant properties, skin moisturizing

## Abstract

This research aimed to synthesize carboxymethyl chitosan (CMCH) from different molecular weights of chitosan including low MW (L, 50–190 kDa), medium MW (M, 210–300 kDa) and high MW (H, 310–375 kDa) on the antioxidant and moisturizing properties. The L-CMCH, M-CMCH and H-CMCH improved the water solubility by about 96%, 90% and 89%, respectively when compared to native chitosan. Higher MW resulted in more viscous of CMCH. For antioxidant properties, IC_50_ values of DPPH and ABTS radical scavenging activity for L-CMCH were 1.70 and 1.37 mg/mL, respectively. The L-CMCH had higher antioxidant properties by DPPH and ABTS radical scavenging assay and FRAP. The moisturizing properties on pig skin using a Corneometer^®^ showed that 0.5% H-CMCH significantly presented (*p* ≤ 0.05) greater moisturizing effect than that of untreated-skin, distilled water, propylene glycol and pure chitosan from three molecular weights.

## 1. Introduction

Chitosan was generally considered in the way that it has low toxicity, biodegradable, accelerates wound-healing, antibacterial properties and gel-forming properties [1]. Chitosan is cheap and inexhaustible material with numerous applications in cosmetics, pharmaceuticals, nourishment science and biotechnology [2,3]. The uses of chitosan are restricted because of its insolubility at neutral or basic region. Hence the solubility of chitosan must be improved. Carboxymethylation is a chemical modification which can improve water solubility. The water solubility properties and applications of carboxymethyl chitosan (CMCH) strongly depended on its structural characteristics, the average degree of substitution (DS), the position of the carboxymethylation (grafting to amino or hydroxyl groups) and the average number of hydroxyl groups substituted by carboxymethyl groups [4]. The CMCH is prepared by the replacement of –OH groups of chitosan with –CH_2_COOH groups with the alternative functional groups such as *O*-, *N*- and *N,O*- carboxymethyl chitosan [2]. Substitution of *N*- and *O*-carboxymethyl chitosan derivatives take place when chitosan reacts with monohalocarboxylic acids using different reaction conditions to control the selectivity of reaction such as temperature and ratios of chitosan, pH as well as monochloroacetic acid. Promotion of *O*-substitution occurs when the reaction is carried out at low temperature such as 0–10 °C [5,6], but *N*-substitution is dominated at high temperature [7]. The optimal reaction temperature of N-CMCH synthesized from chitosan was 90 °C [8]. The solubility of cellulose derivatives did not only depend on the DS, but also on the distribution of the substituents for glucose units along the cellulose chain [9]. The –COOH and–NH_2_ groups replacement indicate capacity of the chemical modifications to improve their physical properties [3]. CMCH is dissolvable in a wide pH range with several advantages and low harmfulness [10]. CMCH not only has a good solubility in water, but also has unique chemical, physical and biologic properties such as high viscosity, large hydrodynamic volume, biocompatibility, good ability to form films, fibers and hydrogels [11,12,13]. Hence, it was widely utilized in numerous biomedical fields, for example, a moisture-retention agent, wound dressing agent, artificial bone and skin, blood anticoagulant and as a component in different drug delivery [14]. Chitosan and CMCH were investigated for coating and film forming abilities to extend product shelf life. The effects of different chitosan types and molecular sizes on properties of CMCH films to plastic replacement were also studied [15]. Zhang et al. [16] found that chitosan modification could improve the antioxidant activity by addition of quaternium on amino groups. Ying et al. [17] prepared various Schiff base typed chitosan saccharide derivatives to enhance the ability of DPPH scavenging radical and also water solubility in comparison to native chitosan. Moreover, antioxidant activities of *N*-carboxymethylchitosan oligosaccharides with different DS (0.28, 0.41 and 0.54) were also evaluated by the scavenging of DPPH radical, superoxide anion and the determination of reducing power. The increase in DS of *N*-CMCH resulted in decreased DPPH radical scavenging activity with increased reducing power [18].

The antioxidant activities of chitosan and CMCH are evidence that the active hydroxyl and amino groups within the polymer chains may participate in free radical scavenging which were varied with MW [17]. Zhao et al. [19] reported that CMCH was a better antioxidant than native chitosan, especially in terms of its reducing power, scavenging ability towards DPPH and superoxide radicals as well as chelating ability of ferrous ions. In case of native chitosan, the moisture-absorption and moisture-retention capacities of chitosan depended on the MW and DS. The ability to absorb moisture increased when the MW was decreased [20]. Humectant property of chitosan improved with increasing MW. For the CMCH, Jimtaisong et al. [21] reported that MW and DS could also affect the exhibitions of the moisture-retention capacity of CMCH. Water-holding capacity of CMCH is related to the presence of positive electrical charges and high molecular weight that facilitate adherence onto the skin when implemented as a skin moisturizer. Muzzarelli et al. [22] revealed that 0.25% CMCH solution was comparable with 20% propylene glycol in terms of moisture-retention capacity with equivalent viscosity to hyaluronic acid (HA), a compound with excellent moisture-retention property. Furthermore, moisture absorption and moisture retention capacities of CMCH could also be significantly improved by utilizing higher MW with the presence of intermolecular hydrogen bonds within molecular chains. Gel formation resulting from addition of CMCH as hydrating agent in cosmetics is also ideal for the skin as it asserts positive feeling of the customers. In cosmetic products, humectants are used to increase the amount of water in the top layers of the skin. The activity of humectant polymers depends on cationic charges, molecular weight and hydrophobicity of polymer. The positively charged ions facilitate neutralization of negatively charged ions on the skin [23].

However, the antioxidant and moisturizing properties of CMCH prepared from different molecular weights of chitosan have not yet been investigated. Therefore, this research aimed to synthesize CMCH from different molecular weights of chitosan including low MW (L, 50–190 kDa), medium MW (M, 210–300 kDa) and high MW (H, 310–375 kDa) and characterized their respective antioxidant and moisturizing properties.

## 2. Materials and Methods

### 2.1. Materials

Three different molecular weights of chitosan including low MW (L, 50–190 kDa), medium MW (M, 210–300 kDa) and high MW (H, 310–375 kDa) with degree of deacetylation above 90% were obtained from Ta Ming Enterprises Co., Ltd.; Samutsakon, Thailand. Ethanol, methanol, isopropanol, sodium hydroxide and glacial acetic acid were purchased from RCI Labscan (Bangkok, Thailand). Monochloroacetic acid was obtained from Sigma-Aldrich (Darmstadt, Germany). All other reagents were of analytical grade.

### 2.2. Synthesis of CMCH

CMCH was synthesized by following method of Tantala et al. [14]. Chitosan flake was grounded and sieved to obtain particle size under 60-mesh (Endecotts, UK). The chitosan (25 g) was suspended in 50% (*w/v*) sodium hydroxide solution (400 mL) and 100 mL of isopropanol was added and mixed well at 50 °C for 1 h. Monochloroacetic acid (50 g) was dissolved in isopropanol (50 mL), gradually dropped into the reaction for 30 min and the system was allowed to continuously react at 50 °C for 4 h. The reaction was stopped by adding 70% (*v/v*) methanol. The pH of the sample was later adjusted to 7.0 by 1% (*v/v*) glacial acetic acid. From that point, the solid was separated and washed in 70–90% ethanol for desalting and dried in a hot air oven (Binder, Germany) at 80 °C for 12 h. The mass yield of CMCH was calculated using Equation (1).

(1) $$\mathrm{Yield}\;(\%)=\frac{\mathrm{chitosan}\;(g)\;-\;\mathrm{CMCH}\;(g)\;}{\mathrm{chitosan}\;(g)}\;\times \;100$$

### 2.3. Moisture Content, pH and Viscosity Measurement

Moisture content was determined according to the Association of Official Analytical Chemists (AOAC) standard method no. 930.15 [24]. The pH values were measured by a pH meter (FiveEasy F20, Metter Toledo, Switzerland). The viscosity of 1% (*w/v*) solution of chitosan and CMCH were estimated by Brookfield viscometer (DV-II, Brookfield Engineering Labs Inc., Stoughton, MA, USA) using a spindle No. 28 at 100 rpm.

### 2.4. Water Solubility of CMCH

The water solubility of CMCH samples at 25 °C was tested by using the method of Rachtanapun et al. [15]. After addition of 0.3 g samples (initial dried weight) into 10 mL water (3% w/v), the solutions were filtered with Whatman filter paper No. 4 (Sigma-Aldrich, Germany) which was previously dried at 105 °C for 24 h before use. The mass of dried CMCH residues was obtained by weight difference to obtain final dry weight. The tests were performed in triplicate to detect random error and the solubility was determined using Equation (2).

(2) $$\mathrm{Water}\;\mathrm{solubility}\;(\%)=\frac{\mathrm{initial}\;\mathrm{dried}\;\mathrm{weight}\;\mathrm{of}\;\mathrm{CMCH}\;(g)\;-\;\mathrm{final}\;\mathrm{dried}\;\mathrm{weight}\;\mathrm{of}\;\mathrm{CMCH}\;(g)}{\mathrm{initial}\;\mathrm{dried}\;\mathrm{weight}\;\mathrm{of}\;\mathrm{CMCH}\;(g)}\;\times \;100$$

### 2.5. FTIR Analysis

The FTIR spectra of chitosan and CMCH were obtained using a Fourier transform infrared spectrometer (Frontier, PerkinElmer, Waltham, MA, USA). All spectra were recorded in the range of 500–4000 cm^−1^ as described by Surin et al. [25].

### 2.6. Antioxidant Properties

The stock solution of L, M and H (stock 5-mg/mL in 0.2% (*v/v*) acetic acid), L-CMCH, M-CMCH and H-CMCH (stock 5-mg/mL in distilled water) at different concentrations of 1, 2, 3, 4 and 5-mg/mL were prepared and used for DPPH, ABTS and FRAP assays.

#### 2.6.1. DPPH Radical Scavenging Assay

The ability of antioxidants to scavenge the 2,2-diphenyl-1-picrylhydrazyl (DPPH) free radical was completed by modified method of Hu et al. [26]. After that, 100 µL of the stock samples (as described above) were blended with 100 µL of 0.2-mM DPPH reagent (Sigma-Aldrich, Singapore) and incubated at 25 °C for 30 min in the dark. Absorbance was measured at 517 nm in a 96-wells microplate reader (SpectraMax^®^ i3x, Molecular Devices, San Jose, CA, USA). The radical scavenging activity of the sample was calculated based on the gallic acid (Sigma-Aldrich, Schnelldorf, Germany). Results were expressed as milligram gallic equivalent per gram of sample (mgGAE/g sample). The percentage of DPPH radical scavenging activity can be calculated as shown in Equation (3) before plotting of IC_50_ against respective concentration.

DPPH radical scavenging activity (%) = [(A_517_ control − A_517_ sample)/A_517_ control] × 100 (3)

#### 2.6.2. ABTS Radical Scavenging Assay

The 2,2-Azino-bis-(3-ethylbenzothiazoline-6-sulfonic acid) (ABTS) radical scavenging activity was conducted according to method described by Xie et al. [27]. ABTS (Sigma-Aldrich, Singapore) reagent was freshly prepared by mixing 8 mL of 7-mM ABTS stock solution with 12.5 mL of 2.45-mM potassium persulfate (Sigma-Aldrich, Singapore). ABTS powder and potassium persulfate powder were individually dissolved with water to the required concentration and then combined together in a bottle. After 16 h of incubation in the dark at 25 °C, the resultant dark blue color of ABTS reagent solution was diluted with ethanol until the absorbance reading reached 0.7 ± 0.2. The solution of L, M, H, L-CMCH, M-CMCH and H-CMCH were prepared as described previously in Section 2.6.1. Each sample solution (0.5 mL) was mixed with 1.0 mL of ABTS stock solution and incubated for 6 min in the dark. Absorbance was measured at 734 nm in the 96-well microplate reader. The ABTS radical scavenging activity was expressed as milligram gallic equivalent per gram of sample (mgGAE/g sample). The percentage of ABTS radical scavenging activity can be calculated as shown in Equation (4) with plotting of IC_50_ against respective concentration.

ABTS radical scavenging activity (%) = [(A_734_ control − A_734_ sample)/A_734_ control] × 100 (4)

#### 2.6.3. FRAP Assay

The ferric reducing antioxidant power (FRAP) assay was carried out according to the technique of Woranuch et al. [28]. The FRAP reagent was prepared by mixing 25 mL of 0.3-M acetate buffer (pH 3.6), 2.5 mL of 4,6-tripyridyl-s-triazine (TPTZ) (Sigma-Aldrich, Schnelldorf, Germany) solution in 40-mM HCl (RCI Labscan, Bangkok, Thailand) and 2.5 mL of 20-mM ferrous sulfate (Loba Chemie, India). Thus, 50 µL of samples were mixed with 950 µL of FRAP reagent and incubated in dark for 30 min. Absorbance was measured at 593 nm in 96-well microplate. The ferric reducing antioxidant power of sample was determined based on the ferrous sulfate (Merck, Darmstadt, Germany). Results were expressed as ferrous sulfate equivalent antioxidant capacity, with µmol Fe^2+^/g sample.

### 2.7. Moisturizing Properties on Pork Skin

The skin moisturizing of the 0.5% (w/v) L, M, H, L-CMCH, M-CMCH and H-CMCH solutions were examined on pork skin and compared with untreated-skin, water and propylene glycol. The pork skins were prepared from back side of the pig ear obtained from three different market sources including the Mae Hia fresh market, the Ton Payom fresh market and the Hangdong fresh market (Chiang Mai, Thailand). The samples were washed and cleaned with removal of the fat layer prior to cutting into 3 × 3 cm. Each sample (100 µL) was applied on the skin surface. The skin without any substance was used as a control. The skin moisturizing was measured before applying on samples and after application at 0, 15 and 30 min intervals using Corneometer^®^ (Courage + Khazaka Electronic, Germany). Before applying the sample and recording the parameter, the pig skins were kept at 25 °C for 30 min. This method was adapted from Kassakul et al. [29]. The degree of skin moisturizing (%) was tested in triplicate to detect random error and calculated using Equation (5).

(5) $$\mathrm{Degree}\;\mathrm{of}\;\mathrm{skin}\;\mathrm{moisturizing}\;(\%)=\frac{\;\mathrm{after}\;\mathrm{applying}\;-\;\mathrm{before}\;\mathrm{applying}}{\mathrm{before}\;\mathrm{applying}}\times 100$$

### 2.8. Statistical Analysis

All data were analyzed by one-way ANOVA. Mean separation was performed by Duncan’s multiple range tests with significance level (*p* ≤ 0.05). Statistical analyses were performed with the SPSS 17.0 (SPSS, Inc.; IBM Corp.; Chicago, IL, USA).

## 3. Results and Discussion

### 3.1. Effect of CMCH Synthesis

CMCH was prepared at three different molecular weights of chitosan (L, M and H). The yield, moisture content, water solubility, viscosity and pH of chitosan products (L-CMCH, M-CMCH and H-CMCH) were reported in Table 1. L-CMCH had the highest yield, water solubility and viscosity, while moisture content and pH of L-CMCH, M-CMCH and H-CMCH were not significantly different (*p* > 0.05) among the range of 6.36–6.87% and 7.27–7.33%, respectively. The solubility is a significant property of CMCH that measures their resistance to water. Table 1 shows water solubility of the L-CMCH M-CMCH and H-CMCH which indicates the significant effect (*p* ≤ 0.05) of larger MW on decreased water solubility. The decreasing trend was 96.87% for L-CMCH, 90.06% for M-CMCH and 89.49% for H-CMCH compared to the L, M and H. The solubility and conformation of CMCH happens from the deacetylation, pH and MW of native chitosan. The solubilization process of CMCH related to functionalized polymers, different types of chemical and physical interactions such as hydrogen bonds, hydrophobic interactions and van der Waals forces. high water solubility suggests that CMCH is moisture absorption and more helpful ability to bind with water than chitosan. higher solubility is due to forming hydrogen bonding with carboxylic groups of CMCH with water molecules. This causes the hydrated water molecules that around the chain of CMCH are more than that surrounding the chitosan chains, resulting in higher water solubility [30]. This results also are consistent with the report of Siahaan et al. [31] who found that the temperature and NaOH concentration affected to CMCH synthesis. The interactions between NaOH and monochloroacetic acid resulted in reduced CMCH forming and lower solubility. The mitigation in solubility may stem from the loss of free amino-functional groups that enhance hydrophobic nature of the compounds [32]. The greater solubility also corresponded to the decrease in viscosity L-CMCH and M-CMCH are slightly different, but H-CMCH requires significantly higher viscosity. This could be explained that CMCHs with chains longer or higher MW were contributing to the gel.

**Figure 1:**
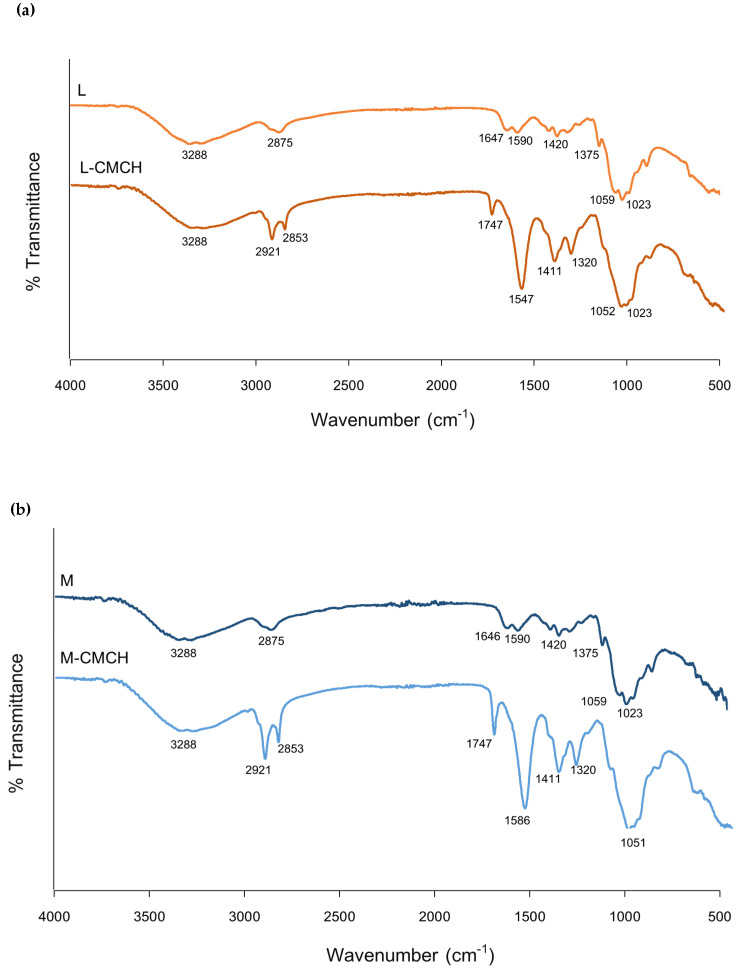

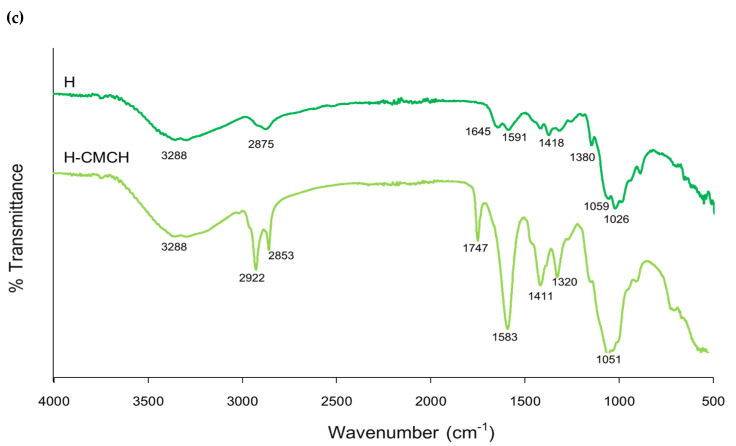
FT-IR spectra of (**a**) L and L-CMCH; (**b**) M and M-CMCH; (**c**) H and H-CMCH.

FTIR spectra of chitosan and CMCH are presented in Figure 1. The essential characteristic peaks of chitosan are at 3288 cm^−1^ (O–H stretch), 2875 cm^−1^ (C–H stretch), 1591–1645 cm^−1^ (N–H bend), 1059 cm^−1^ (bridge-O stretch) and 1023 cm^−1^ (C–O stretch) [2]. For CMCH, the spectrum was different from the spectrum of chitosan (Figure 1 and Table 2). The IR spectrum of CMCH showed the intrinsic peak at 1747 cm^−1^, the most visible difference was the appearance of a new peak which belonged to C = O stretching vibration (amide I) Putra et al. [33] identified C = O peak on CMCH whose wave number could be 1600–1850 cm^−1^, 1660–1680 cm^−1^ and also 1606 cm^−1^. The CMCH showed the disappearance of the–NH_2_ associated band at 1647 cm^−1^ which could be associated with characteristic vibration deformation of the primary amine N–H and the combination N–H peak with new peak at 1583. The appearance of some new intensive peaks at 2922–2853 and 1583 cm^−1^ could be attributed to the methyl groups and the long carbon segment of the quaternary ammonium salt [34]. Compared to the peaks of chitosan, the new bands at 1583 cm^−1^ and 1411 cm^−1^ corresponded to the carboxy group (overlapped with N–H bending) and–CH_2_COOH group, respectively. The intense spectrum of CMCH indicating carboxymethylation on both the amino and hydroxyl groups of chitosan [2]. Characteristic peaks of the first C–O and the second C–O groups between 1052 and 1024 cm^−1^ (C–O stretch) did not change. It was confirmed that chitosan was converted to CMCH by new transmission peaks of -COO groups at 1583 and 1747 cm^−1^. These new -COO groups enhanced hydrophilic properties of the CMCH which enhanced solubility of the compound.

### 3.2. Antioxidant Properties

**Figure 2:**
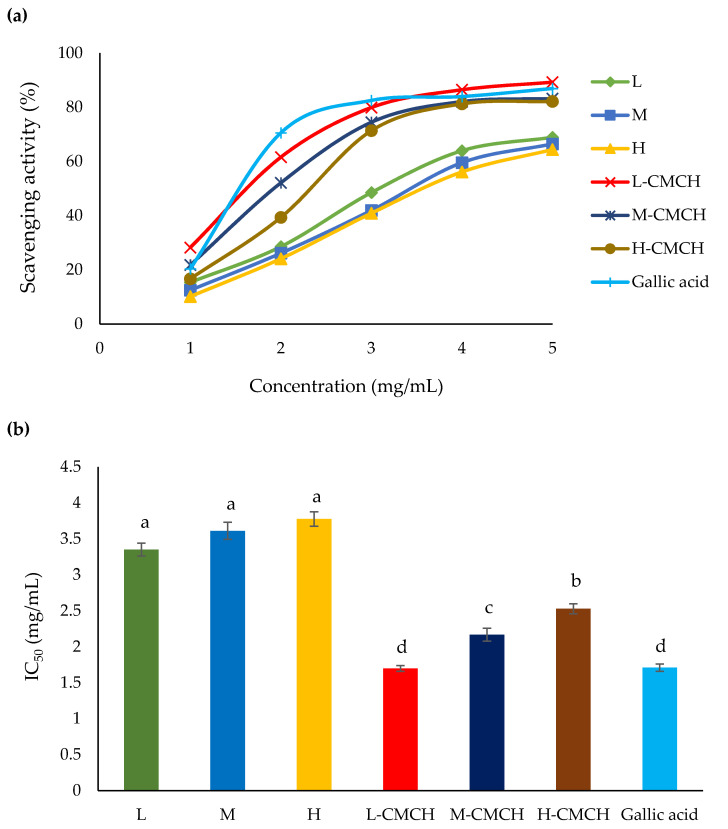
(**a**) DPPH radical scavenging activity (%) and (**b**) IC_50_ of L, M, H, L-CMCH, M-CMCH and H-CMCH. Different letters (a–d) indicate significant difference between treatments (*p* ≤ 0.05).

**Figure 3:**
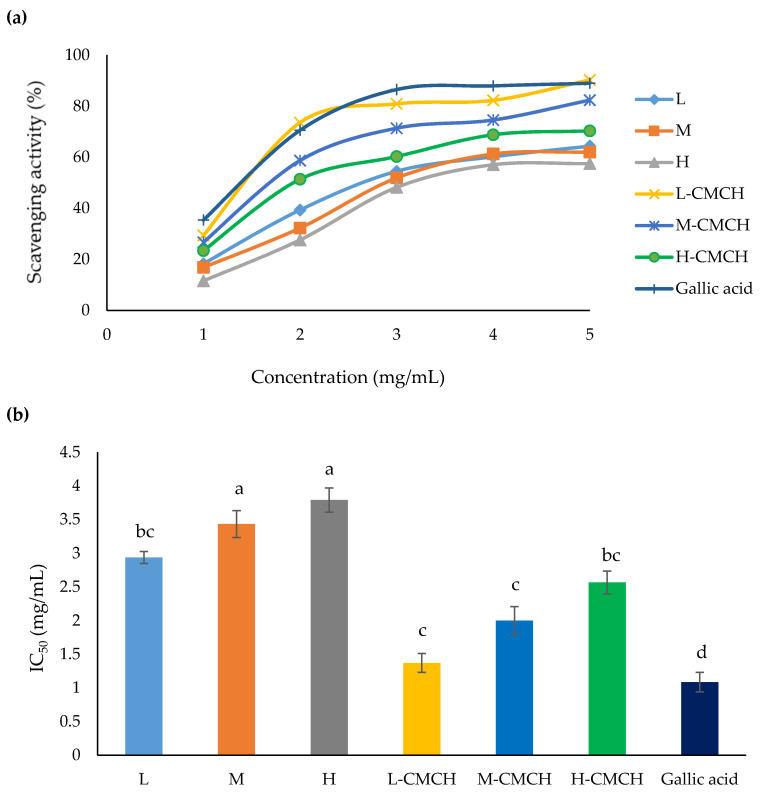
(**a**) ABTS radical scavenging activity (%) and (**b**) IC_50_ of L, M, H, L-CMCH, M-CMCH and H-CMCH. Different letters (a–d) indicate significant difference between treatments (*p* ≤ 0.05).

The results from DPPH assay of L, M, H, L-CMCH, M-CMCH and H-CMCH are shown in Figure 2. L-CMCH showed the highest (*p* ≤ 0.05) scavenging activity. The DPPH scavenging activities of L-CMCH, M-CMCH and H-CMCH were higher than those of L, M and H. IC_50_ values of DPPH and ABTS radical scavenging activities of L-CMCH were 1.70 and 1.37 mg/mL, respectively. However, no significant differences in DPPH scavenging potential were found among the L, M and H. Younes et al. [35] also found that IC_50_ value was determined between 1.62- 2.20 mg/mL for shrimp chitosan (*Metapenaeus monoceros*) at different concentrations (0–5 mg/mL). The DPPH radicals scavenging ability of chitosan and its derivatives (CMCH) increased as the concentration increased [36]. This is probably due to the relatively poor hydrogen-donating ability of chitosan that prevent chain breaking [37]. Some studies suggested that DPPH radical scavenging of chitosan increased with decreasing MW [38]. Again, it is confirmed that the CMCH have strong antioxidant activity, which is dependent on the particle size [34]. In addition, chitosan chains possess active hydroxyl and amino groups that can react with free radicals [39]. Scavenging activity of chitosan is related to the extent of reaction between free radicals and protonated amino groups [18]. Our results indicated that the DPPH scavenging ability of CMCH synthesized with low, medium or high MW was higher than that of pure chitosan from three MW. Elbarbary & Mostafa [40] also confirmed that the antioxidant activities of CMCH could be enhanced by decreasing MW of CMCH. high MW can contribute to a more compact structure and relatively stronger effect of intramolecular hydrogen bond. The antioxidant activity for ABTS radical was similar to those of DPPH assay even though ABTS radicals are more reactive than DPPH radicals [19]. Hence, these showed that antioxidant activity is expanded with decreasing MW with L-CMCH, M-CMCH and H-CMCH, respectively, compared to the L, M and H Figure 3.

**Figure 4:**
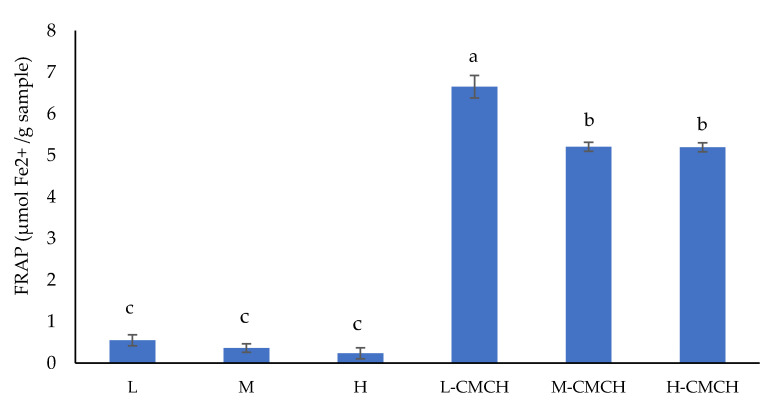
Ferric reducing antioxidant power (FRAP) of L, M, H, L-CMCH, M-CMCH and H-CMCH. Different letters (a–c) indicate significant difference between treatments (*p* ≤ 0.05).

FRAP assay in Figure 4 revealed the variation of antioxidant capacity with corresponding concentration levels [41]. In similar manner of DPPH assay, control could slightly reduce ferric to ferrous ions. In this assay, L-CMCH had the highest (*p* ≤ 0.05) ability to reduce ferric to ferrous ion [42], followed by M-CMCH and H-CMCH, while L, M and H showed the lowest ability (*p* ≤ 0.05). The replacement of -NH groups by -COO groups in the CMCH structure was previously reported to be beneficial not only to level of solubility, but also antioxidant activities [43]. Although some studies suggested the effects of molecular weights to FRAP antioxidant activities [44], such effect was not evident in current study.

### 3.3. Skin Moisturizing Properties

**Figure 5:**
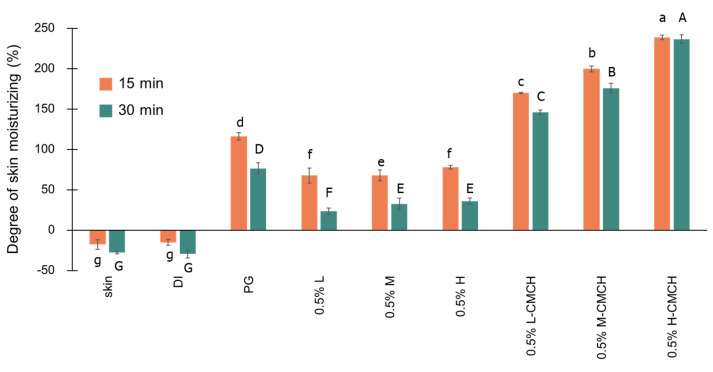
Degree of skin moisturizing (%) as affected by time (15 and 30 min) and different treatments (skin, DI, PG, L, M, H, L-CMCH, M-CMCH and H-CMCH) on pork skin Different lowercase letters (a–g) indicate significant differences between solutions at 15 min and different uppercase letters (A–G) indicate significant differences between solutions at 30 min.

The degree of skin moisturizing indicates the water-holding capacity of the skin which can be tested by the Corneometer method. The Corneometer^®^ measures the changes of electrical capacitance related to the moisture contents of the skin before and after applying the solutions [29]. The degree of skin moisturizing of the L, M, H, L-CMCH, M-CMCH and H-CMCH solutions were examined on pork skin and compared with untreated skin, water and propylene glycol at 15 and 30 min as presented in Figure 5. The effect degree of moisturizing on time at 15 and 30 min showed that the degree of skin moisturizing of solutions decreased with increasing time after applying solutions, except 0.5% H-CMCH. The degree of skin moisturizing of H-CMCH had no significant difference after applying between 15 and 30 min. Applying H-CMCH solution for 15 and 30 min were the highest degree of skin moisturizing, showing high moisturizing effect (more than 200%). While the degree of skin moisturizing of untreated skin, water propylene glycol, L, M, H, L-CMCH and M-CMCH solutions applying on pork skin for 30 min were significantly decreased from 15 min. This confirms that the H-CMCH solution provided a good moisture absorption. In fact, the skin moisturizing effects appeared to decrease with increasing time due to lack of mechanisms to maintain skin moisturizing and dryness of pork skin cells [45]. The higher molecular weight CMCH also had the superior moisture retention capacity. Kassakul et al. [29] found that 0.2% *Hibiscus rosa sinensis* mucilage as natural ingredient provided good results of skin moisturizing after applying for 30 min by about 130%. The results showed that moisturizing products could increase the water content of the skin while maintaining softness and smoothness [20]. After applying solutions containing different MW of water-soluble CMCH (L-CMCH, M-CMCH, H-CMCH), the moisture content of the skin increased. The mechanism of moisturizing effect is based on the formation of water film of skin surface after dissolution of CMCH and subsequent stage of water evaporation could further prevent water evaporation from the skin [46]. Positive electrical charges and relatively high MW facilitates prolong skin adherence [21]. Our results also showed that H-CMCH decreased the loss of water while elevating skin humidity. The higher apparent viscosity of H-CMCH can improve the stability and enhance skin hydration. In fact, 0.5% H-CMCH was superior to untreated skin, water and propylene glycol in terms of degree of skin moisturizing effect. The higher MW of CMCH also indicates potential for film forming and coating to multilayer of the skin. Subsequently, it could be used in cosmetic preparation with suggested further studies of the testing skin irritation in human subjects.

## 4. Conclusions

Carboxymethyl chitosan (CMCH) was effectively synthesized and characterized by FTIR. The modifications in biologic properties including water solubility, antioxidant properties as well as efficacy of moisturizing property of CMCH were evident. It is clearly seen that higher MW of chitosan and CMCH resulted in lower antioxidant properties but provided greater moisturizing property. The H-CMCH improved the water solubility by about 89%, when compared to chitosan. The higher levels of DPPH, ABTS and FRAP were also detected. The moisturizing effect was at the highest level when 0.5% H-CMCH was applied to pig skin. H-CMCH is an effective water-soluble polymer with high viscosity which could be successfully utilized in pharmaceuticals and cosmetics as emulsion stabilizers and thickening agents. Future work is required to investigate this biopolymer for skin irritation in human subjects.

## Figures and Tables

**Table 1:** Yield, moisture content, water solubility, viscosity and pH of carboxymethyl chitosan (CMCH) with different MW.

CMCH	Yield (%)	Moisture Content (%) ^ns^	Water Solubility (%) *	Viscosity (cP)	pH ^ns^
L-CMCH	41.33 ^b^ ± 0.34	6.87 ± 0.12	96.87 ^a^ ± 0.29	325.74 ^c^ ± 0.32	7.27 ± 0.29
M-CMCH	43.70 ^a^ ± 1.55	6.36 ± 0.51	90.06 ^ab^ ± 3.30	336.83 ^b^ ± 0.16	7.32 ± 0.22
H-CMCH	45.36 ^a^ ± 0.65	6.56 ± 0.60	89.49 ^b^ ± 3.72	360.05 ^a^ ± 0.84	7.33 ± 0.13

* Water solubility (%) of L-CMCH, M-CMCH and H-CMCH indicates the comparison to native chitosan. Different letters (a–c) in each column indicate significant differences (*p* ≤ 0.05). ns means no significant difference.

**Table 2:** Functional groups and wave number (cm^−1^) of chitosan and CMCH.

Functional Groups	Wave Number (cm^−1^)	
	Chitosan	CMCH
NH_2_ association in primary amines, OH association in pyranose ring	3288	3289
CH_2_ in CH_2_OH group	2873	2917
C = O in NHCOCH_3_ group (amide I)	1647	1747
N–H bending (amide II)	1591	1583
CH_3_ in CH_2_OH group	1421	1411
C–N stretching (amide II)	1319	1320
C–O, C–O–C stretching	1023	1052
